# Advancements in tissue engineering for articular cartilage regeneration

**DOI:** 10.1016/j.heliyon.2024.e25400

**Published:** 2024-02-01

**Authors:** Maohua Chen, Zhiyuan Jiang, Xiuyuan Zou, Xiaobo You, Zhen Cai, Jinming Huang

**Affiliations:** a Department of Plastic Surgery, Sichuan Provincial People's Hospital, University of Electronic Science and Technology of China, Chengdu, Sichuan, China; b Department of Rehabilitation Medicine, West China Hospital, Sichuan University, Chengdu, China; c Key Laboratory of Rehabilitation Medicine, West China Hospital, Sichuan University, Chengdu, China

**Keywords:** Articular cartilage injury, Cartilage repair, Cartilage regeneration, Osteoarthritis, Tissue engineering

## Abstract

Articular cartilage injury is a prevalent clinical condition resulting from trauma, tumors, infection, osteoarthritis, and other factors. The intrinsic lack of blood vessels, nerves, and lymphatic vessels within cartilage tissue severely limits its self-regenerative capacity after injury. Current treatment options, such as conservative drug therapy and joint replacement, have inherent limitations. Achieving perfect regeneration and repair of articular cartilage remains an ongoing challenge in the field of regenerative medicine. Tissue engineering has emerged as a key focus in articular cartilage injury research, aiming to utilize cultured and expanded tissue cells combined with suitable scaffold materials to create viable, functional tissues. This review article encompasses the latest advancements in seed cells, scaffolds, and cytokines. Additionally, the role of stimulatory factors including cytokines and growth factors, genetic engineering techniques, biophysical stimulation, and bioreactor systems, as well as the role of scaffolding materials including natural scaffolds, synthetic scaffolds, and nanostructured scaffolds in the regeneration of cartilage tissues are discussed. Finally, we also outline the signaling pathways involved in cartilage regeneration. Our review provides valuable insights for scholars to address the complex problem of cartilage regeneration and repair.

## 1 Introduction

Articular cartilage (AC) injury caused by external violence or degenerative disease often leads to progressive tissue degeneration, resulting in debilitating joint pain, functional impairment, and degenerative arthritis [1,2]. The management of AC injury is one of the most challenging clinical problems faced by orthopaedic surgeons [2]. AC is hyaline cartilage that covers the two opposing bony surfaces that make up a movable joint [3]. The main physiological functions of AC are to transmit load evenly, expand the weight-bearing surface of the joint, reduce contact stress and cushion shock, and provide a smooth interface with low friction and low wear for joint activities [4]. Consequently, it is one of the main causes of mobility disorders. However, unlike other tissues, AC has unique properties: it is a special connective tissue in which chondrocytes secrete proteoglycans and form a cartilage matrix with type II collagen (COL2), with no blood vessels, nerves or lymphatic vessels [5]. Therefore, repairing AC injury remains a significant clinical obstacle, and developing effective regenerative strategies is critical.

Traditional treatment options for AC injury, including conservative medical therapy [6], physical therapy [7], exercise therapy [8], knee injections [9], and surgical intervention [10], have certain limitations in achieving long-lasting integrity and durability at the regenerative medicine level [11,12]. Conservative medical treatment mainly focuses on symptom management and can only provide temporary relief [13]. Physical therapy or exercise therapy should be conducted under the guidance of trained rehabilitation professionals to ensure proper technique and minimize the risk of exacerbating AC injury due to incorrect methods [7]. Knee injection therapy carries potential risks, including short duration of action, the need for frequent repeated injections, variability in individual efficacy, and the risk of infection [14]. Surgical intervention is an invasive procedure associated with certain risks and is typically reserved for advanced or end-stage cases [2]. These limitations have sparked a growing expectation for research on cartilage regeneration.

Tissue engineering is an emerging field that combines principles from biology, engineering, and materials science. Its advantages lie in its ability to promote tissue regeneration and repair, provide personalized treatment strategies, and reduce rejection reactions and donor dependence [15]. The core technology involves the use of scaffold materials to provide structural support and achieve therapeutic effects by stimulating the body's self-healing capabilities or transplanting a small number of tissue cells cultured and expanded in vitro to form new viable tissues in vivo [16]. These characteristics make tissue engineering the most promising field for addressing AC regeneration [17].

**Fig. 1:**
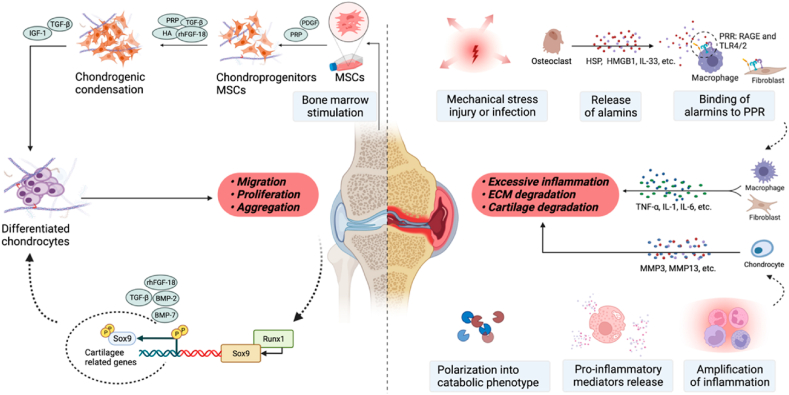
Schematic depicting the mechanisms of cartilage degradation and cartilage repair. When cartilage damage occurs due to various causes, an inflammatory environment ensues, leading to chondrocyte death and hypertrophy, abnormal differentiation of mesenchymal stem cells (MSCs), breakdown of the extracellular matrix (ECM), and cartilage degradation. This results in the formation of fibrocartilage with suboptimal mechanical properties. MSCs play a crucial role in bone regeneration through several processes, including migration, proliferation, aggregation, response to inflammation, and differentiation, achieved by releasing diverse mediators. Following bone marrow stimulation, various biological interventions influence MSCs' chondrogenic differentiation, Sox-9 expression, cartilaginous ECM production, and synthesis and retention of ECM within the articular cartilage. Terms: PDGF: Platelet-derived growth factor; PRP: Platelet-rich plasma; TGF-β: Transforming growth factor β; rhFGF-18: Recombinant human fibroblast growth factor 18; HA: Hyaluronic acid; IGF-1: Insulin-like growth factor; BMP-2: Bone morphogenetic protein 2; BMP-7: Bone morphogenetic protein 7; TNF-α: Tumor necrosis factor α; HSP: Heparan sulfate proteoglycans; HMGB1: High mobility group protein 1; TLR: Toll-like receptor; IL: Interleukin; MMP: Matrix metalloproteinase; PRR: Pattern-recognition receptors.

The field of tissue engineering encompasses three key elements: seed cells, scaffold material, and cytokines [18]. Seed cells for cartilage tissue engineering can be categorized into two main types: chondrocytes and cells with the potential to differentiate into chondrocytes [[19], [20], [21]]. Scaffold materials play a crucial role in providing structural support for cell attachment, migration, and tissue formation [22]. Cytokines, on the other hand, replicate the in vivo conditions and regulate important factors such as cell proliferation and differentiation [23,24] (Fig. 1).

In this review, we briefly introduce the structure and function of cartilage, followed by a focused discussion on the research advancements related to the three key elements mentioned above. Subsequently, we review the regenerative strategies for repairing AC. Finally, the future trends and challenges in the application of bioengineering techniques for treating AC are discussed.

## 2 Method

A PubMed search for cartilage defects as well as chondrogenesis was conducted using the Boolean search string: (articular cartilage) AND (regeneration) AND (tissue engineering). The literature search included all studies published in English between 2013 and 2023. Based on this search, 1581 articles were found on October 2023. Articles including new materials or approaches of cartilage regeneration were included. We first assessed the content of retrieved articles based on titles and abstracts, and then independently assessed the full text for inclusion in our review.

## 3 Articular cartilage: structure and function

AC consists of a solid phase, collagen fibers and proteoglycans, and a liquid phase, water, and electrolytes. AC is rich in ECM, mainly including COL2, PG, and HA. The chondrocytes were distributed among the collagen fibers [25,26]. Anatomically and functionally, AC has four distinct zones: the superficial, transitional, deep, and calcified layers. Each region is characterized by a specific composition of the ECM, unique mechanical properties, and a specific form of cellular organization. The superficial zone contains high levels of COL2 and low levels of glycosaminoglycan. The COL2 content in the transition zone was lower, but the glycosaminoglycan concentration was higher. The content of glycosaminoglycan was the highest and that of COL2 was the lowest in the deep zone. The calcified cartilage zone contains high levels of COLX and integrates the cartilage into the subchondral bone [27]. It has been shown that COL2 content gradually decreases with the gradient from the superficial to the deep layer, while COLX and proteoglycan content increase [28,29]. AC lacks blood vessels, nerves, and lymphatic vessels, which can obtain material, energy, and information transmission by mechanical loading on chondrocytes to maintain the normal physiological state of AC [30].

AC makes the morphology of the joint head and fossa more suitable, its surface is smooth, and there is little synovial fluid between the articular surface, which makes the movement more flexible [31]. Moreover, due to the flexibility of the cartilage, it can withstand the load, slow down the vibration and prevent potentially destructive stress from concentrating on the joint [32]. The tissue composition of AC reflects its mechanical properties. The repair of AC requires full consideration of the biomechanical functional characteristics and mechanobiological laws of AC, which is a major challenge for reconstructing the structure of natural cartilage in vivo and in vitro [33].

## 4 Seed cells for articular cartilage regeneration

### 4.1 Types of seed cells used in tissue engineering

#### 4.1.1 Autologous chondrocytes

Autologous chondrocyte transplantation (ACT) involves the implantation of reparative chondrocytes to the site of cartilage defects to promote repair [34]. The initial generation of ACT technology utilized periosteum sealing. However, due to limitations such as thinness, susceptibility to rupture, and hypertrophy, researchers later replaced periosteum with collagen membrane for sealing and fixation of chondrocytes leading to the second generation of ACT technology [35]. Although ACT has shown satisfactory long-term clinical outcomes, it does have certain drawbacks. For instance, there is a risk of chondrocyte leakage from the graft area, and the distribution of cells within the graft area can be uneven due to factors such as gravity, resulting in an uneven regenerated cartilage surface [35,36]. Prior to transplantation, chondrocytes undergo dedifferentiation in monolayer culture in vitro. Moreover, the clinical application of autologous chondrocyte transplantation still faces challenges such as high cost, technical complexity, and the need for joint incisions [37,38].

The third generation of ACT primarily focuses on improving the delivery of chondrocytes. Chondrocytes are precombined with a carrier and subsequently implanted into the cartilage defect area. This approach allows for the prior fixation of cultured cells, effectively enhancing the postsurgery survival rate of chondrocytes [39]. However, it does have limitations, including the limited availability of chondrocytes sources and the requirement for two surgical procedures.

#### 4.1.2 Mesenchymal stem cells

Mesenchymal stem cells (MSCs) are multipotent stem cells with self-renewal and chondrogenic differentiation capabilities. MSCs play a crucial role in local repair and metabolism regulation, making them an ideal cell source for cartilage regeneration [[40], [41], [42]]. MSCs can be derived from various autologous tissues, including bone marrow stem cells (BMSCs), adipose tissue stem cells (ADSCs), umbilical cord mesenchymal stem cells (UCMSCs), synovial tissue stem cells (SDSCs) [43,44]. However, the therapeutic efficacy of MSCs is limited due to challenges such as inadequate migration to the injury site and poor engraftment site attachment [[45], [46], [47]]. Therefore, measures are required to enhance the migratory capacity of MSCs and accelerate cartilage regeneration.

Exosomes (Exos), secreted by cells and ranging in size from 30 to 200 nm, have gained attention for their ability to carry information between cells and overcome limitations associated with cell transplantation, such as immunogenicity and tumorigenicity [[48], [49], [50]]. Among various cell-derived Exos, MSC Exos have shown immunomodulatory and regenerative functions [51]. Numerous studies have demonstrated the growth-enhancing, anti-inflammatory, anti-apoptotic, and wound healing effects of MSC Exos in various in vitro and in vivo models [51]. For example, MSCs-derived Exos mediate repair in rat model of arthritis characterized by early suppression of pain and degeneration, reduction of inflammation, subsequent sustained proliferation, gradual improvement of matrix expression and subchondral bone structure, which maintains overall joint homeostasis [52].

#### 4.1.3 Induced pluripotent stem cells

Induced pluripotent stem cells (iPSCs) are reprogrammed somatic cells that possess developmental pluripotency similar to embryonic stem cells [53]. They can be generated from various cell types such as fibroblasts, cord blood cells, and peripheral blood monocytes [54,55]. The reprogramming process involves the activation of four transcription factors - Oct4, Sox2, *c*-Myc, and Klf4, or their variants - in mouse and human somatic cells to convert them into iPSCs [56]. Recent studies have identified specific growth factors [57], such as BIX-01294 and BayK8644s, as well as compounds [58] such as AZI2, a DNA methyltransferase inhibitor, and valproic acid, which can improve the efficiency of iPSC induction.

iPSCs possess unique properties of pluripotency and self-renewal, enabling them to differentiate indefinitely into various cell types, making them valuable for disease modeling and regenerative medicine [53,59]. However, bioengineered grafts based on iPSCs are currently limited to laboratory-scale production and for scientific purposes [60]. Both MSCs and iPSCs hold promise in disease modeling, tissue engineering, and personalized therapy [53]. However, most iPSC generation methods involve the use of viral vectors, such as retroviruses and lentiviruses [61], which may randomly integrate into the host cell genome, leading to genetic instability or interference with normal gene function [62]. Moreover, iPSCs exhibit higher osteogenic differentiation capacity and unlimited self-renewal potential, raising concerns about spontaneous teratoma formation [60,62,63].

One significant advantage of iPSCs is their ability to be produced from different donor categories, including both diseased and healthy individuals, making them clinically applicable without the ethical concerns associated with ESCs [64]. iPSC differentiation protocols are important to obtain cell cultures that are as close as possible to natural chondrocytes [65]. In recent years, many protocols for chondrogenic differentiation have been developed. To reduce the risk of off-target differentiation, researchers have proposed the use of small molecule inhibitors of WNT and MITF [66]. In most studies, TGFβ1 and TGFβ3 as well as BMP2 and BMP4 were used as the main chondrogenic induction factors. They found that transduction of TGFβ1 by lentivirus and co-culture with mature cartilage tissue promoted cartilage formation in vitro and reduced VEGF expression by factors secreted by mature chondrocytes (e.g., metastatin) [67]. In recent years, despite the progress in iPSC chondrogenesis and the reports of chondrogenic differentiation protocols without mentioning tumor and teratoma formation, iPSCs differentiation protocols have not yet been approved for use in clinical trials, and there is still a need for particularly stringent controls regarding the safety of iPSCs [53] (Table 1).

**Table 1:** Cell types for cartilage tissue engineering based on animal experiments.

Cells Type			Modality	Animal models	Administration Method	Carrier	Accessory carrier molecule	Outcome	Limitation	Ref.
**Differentiated cells and** Chondrocytes		Allogenic	Living cells	Rabbit knee cartilage defect model	Intra-articular injection	Scaffold	None	Successfully repairing rabbit cartilage injury	Experimental cells are inbred cells and results may not be extrapolated	[68]
		Allogeneic	Living cells	Rat OA model by monoiodoacetic acid method	Intra-articular injection	None	None	Promoting articular cartilage repair and retarding OA progression	No chondrogenic effect of curcumin alone on MSCs	[69]
		Allogeneic	Living cells	Mice OA model by transecting the medial meniscotibial ligament	Intra-articular injection	None	miR-410-3p, HMGB1	miR-410-3p targeted HMGB1 and modulated chondrocytes apoptosis and inflammation	The miR-410-3p regulatory functions in OA remain largely unknown	[70]
**Stem Cells**	BMSCs	Allogeneic	Living cells	Rats osteochondral defect model	Transplanted subcutaneously	Hydrogels	None	Promoting cartilage repair/regeneration	No dose-response and parameters studies of PEMF	[71]
		Allogeneic	Exosome	Rats distal femoral drill-hole growth plate injury model	The defect site injection	Hydrogels	None	Promoting cartilage regener and reducing bone bridge formation	Applicable only if growth plates are present	[72]
		Allogeneic	Exosome	Rat cartilage defect model	The defect site injection	Hydrogels	miR-205-5p	Promoting cartilage regeneration in vivo	NA	[73]
		Allogeneic	Living cells	Rat cartilage defect model	The defect site injection	Hydrogels	None	Improving the chondrogenic differentiation of BMSCs and inducing new hyaline cartilage formation	3D technology application limitations	[74]
		Allogeneic	Living cells	Rat temporomandibular joint arthritis model	Bilateral temporomandibular joint cavity injection	Hydrogel microspheres	TGF-β	Facilitating the repair of irregular cartilage defects	The inability of growth factors to fully penetrate the spheroid core	[75]
		Allogeneic	Living cells	Rabbit femoral distal condyle cartilage defect model	The defect site implantation	Scaffold	miRNA-410	Achieving the overall repair effect on the cartilage surface and the underlying tissue of the surface defect	Lacking the research on miRNA-410 on cartilage repair	[76]
		Allogeneic	Living cells	Rabbit knee cartilage defect model	Intra-articular injection	None	Integrin α10	Chondrocyte-like cells differentiation and cartilage matrix production	No graded cartilage defect healing	[77]
		Allogeneic	Living cells	Rabbit knee cartilage defect model	Subcutaneous implantation	Scaffold	None	Bridging the cartilage injury site with hyaline cartilage	Decreasing the mechanical properties of the ECM	[78]
	ADSCs	Allogeneic	Living cells	Rat OA model by transecting the medial meniscotibial ligament	Intra-articular injection	Hydrogel	None	Capturing the redundant ROS	Lacking large-animal models	[79]
		Allogeneic	Living cells	NA	Injectable	Hydrogel	None	Great cell viability and chondrogenesis improvement	Lacking animal models	[74]
		Allogeneic	Living cells	Rabbit knee cartilage defect model	Intra-articular injection	Hyaluronic acid hydrogel	BMP-14	Promoting cartilage defect repair	No verification of the safety of the BMP-14 gene transfer	[80]
**pluripotent stem cells and** iPSCs		Allogeneic	Living cells	Rat cartilage defect model	Pellet implantation	NA	TGF-β1	Generation of hyaline chondrocytes	Have not optimized the dose and duration of growth factor induction	[55]

Abbreviation: OA: osteoarthritis; MSCs: mesenchymal stem cells; HMGB1: high mobility group protein 1; BMSC: bone marrow stem cells; PEMF: pulsed electromagnetic field; ECM: extracellular matrix; ROS: reactive oxygen species; ADSCs: adipose tissue stem cells; BMP-14: bone morphogenetic protein 14; iPSCs: induced pluripotent stem cells; TGF-β1: Transforming growth factor β 1.

### 4.2 Cell delivery strategies

Physical therapies, such as pulsed electromagnetic field signals and low-intensity pulsed ultrasound, have been employed to enhance the migratory ability and chondrogenic differentiation of MSCs [[81], [82], [83], [84], [85]] External magnetic force has been demonstrated to improve the viability and implantation of MSCs in cartilage defects [86,87]. Pulsed electromagnetic fields in BMSC-loaded hydrogels have shown potential in promoting cartilage healing in animal models [71]. Low-intensity pulsed ultrasound enhances the migration of heterogeneous MSCs at the injury site through the stromal cell-derived factor-1/CXC chemokine receptor 4 signaling pathway [88,89]. Moreover, the presence of low-frequency electromagnetic fields greatly enhances the cartilage repair ability of hydrogels prepared from hydroxyapatite and monomeric COL1 [90]. Mechanical stimulation has also been found to increase Exos expression, and exosomal LncRNA H19 has been shown to enhance proliferation, matrix synthesis, and apoptosis inhibition in chondrocytes [91].

Guan et al. [72] developed a biocompatible GMOCS-Exos hydrogel composed of ECM and BMSC Exos, which promotes cartilage regeneration, reduces inflammation, and modulates the ECM balance. Hypoxia-treated Exos have been found to enhance the proliferation, migration, anabolism, and anti-inflammatory effects of chondrocytes more effectively than Exos under normal oxygen conditions [73]. Icariin has shown promise in accelerating cartilage ECM synthesis, inhibiting degradation, and promoting cartilage-specific gene expression and differentiation [[92], [93], [94]]. The mechanism is related to the increase in aggregation protein, BMP2, and COL2A1 in BMSCs [95] and the activation of the Wnt/s-catenin signaling pathway [96]. In osteoarthritic rats, the combined action of curcumin and BMSCs has been observed to promote AC repair and delayed disease progression [69]. Folic acid and polypeptide thermogels have also demonstrated positive effects on MSC differentiation and chondrogenesis [97].

## 5 Scaffold materials

In cartilage tissue engineering, scaffold materials should possess specific characteristics to support successful regeneration. Key requirements include controlled biodegradation properties with nontoxic degradation products; porous structures allowing for nutrient and metabolite diffusion; support for cell survival, proliferation, differentiation, and extracellular matrix production; anchoring capability in the cartilage defect area with integration into the surrounding tissue; and the ability to provide mechanical support to the new tissue [98].

Scaffold materials for cartilage tissue engineering can be broadly classified into natural matrix materials and synthetic materials [99]. Natural matrix materials encompass bone and cartilage matrix, periosteum, collagen, hyaluronic acid, cellulose, etc. Synthetic materials commonly used include carbon fiber, hydroxyapatite, polylactic acid, polyglycolic acid, sodium alginate gel, etc. [68]. Some of these materials not only serve as carriers for growth factors or cells but also stimulate the growth of host cells and the synthesis of cartilage matrix. Due to differences in biocompatibility, porosity, degradability, and other properties, these materials can have varying effects on chondrocyte growth and proliferation. Natural materials are often utilized in the form of hydrogels, which have a highwater content and can be designed for injectability, facilitating integration with cartilage repair cells. Hydrogel materials offer the advantage of maintaining the rounded AC phenotype without differentiation and are preferred in cellular mechanical signaling studies due to their ability to transmit mechanical signals to internal cells [100,101].

Hyaluronic acid (HA), a crucial component of synovial fluid, provides a microenvironment similar to native cartilage and promotes the early differentiation of MSCs into cartilage by enhancing aggregated proteoglycans and COL2 [102,103]. Martin et al. [104] developed an electrospun cell-free fibrous HA scaffold that delivers specific growth factors, such as SDF-1α and TGF-β3, which promote cartilage repair and improve the recruitment of mesenchymal progenitor cells and matrix deposition. Although several HA scaffolds have been investigated for cartilage repair, they are still in the research process and no material has yet been translated into clinical applications.

Collagen is the most abundant protein in connective tissue, cartilage as well as skin. The biodegradability and low antigenicity of collagen make it an excellent material for tissue engineering. Lim et al. [105] validated the cartilage repair ability by injecting clinical-grade soluble COL1 containing human nasal septum-derived chondrocytes. Compared with collagen injection alone, cell-loaded collagen promoted cartilage repair in rats more favorably. The excellent properties of collagen itself have led many collagen-based products into clinical studies. For example: ？？

The adverse microenvironment in the joint cavity, characterized by the accumulation of reactive oxygen species (ROS) and excessive inflammation, severely affects the biological activity of transplanted stem cells. Epigallocatechin-3-0-gallate (EGCG) is a green tea catechin with potent ROS scavenging and antioxidant properties that has been utilized to protect ADSCs from ROS-mediated death and bioactivity inhibition. Li et al. [79] prepared a long-acting injectable hydrogel composed of EGCG and HA, which consistently and efficiently captures excess ROS. Intra-articular injection of this hydrogel loaded with ADSCs significantly polarized synovial macrophages toward the M2 phenotype, decreased the expression of proinflammatory cytokines (e.g., IL-1β, MMP-13, and TNF-α), and promoted cartilage matrix formation.

Chondroitin sulfate (CS) is commonly employed as a scaffold material in cartilage tissue engineering, although the rapid degradation of pure CS scaffolds poses a challenge [74]. Li et al. [74] discovered that rat ADSCs in CS-SH/HB-PEG hydrogel exhibited good cell viability, favorable chondrogenesis, and reduced inflammatory response of stem cells, which synergistically promoted cartilage repair. Furthermore, the addition of CS-SILY molecules to COL1/2 hydrogels encapsulated with MSCs greatly enhanced the cartilage repair potential [106].

Chitosan (CH), structurally similar to cartilage ECM glycosaminoglycan, plays a role in helping hyaline cartilage resist shear and compression forces [68]. Through further research, CH can be modified to obtain different properties, such as improved mechanical properties, osteoinductivity, and reduced immune response, by altering the molecular mass and degree of deacetylation. Zhou et al. [107] developed catechol-modified chitosan (CS–C) hydrogels catalyzed by horseradish peroxidase/hydrogen peroxide. These biocompatible CS-C hydrogels promote the proliferation and chondrogenic differentiation of BMSCs in vitro. For in vivo cartilage defect repair, BMSC-loaded CS-C hydrogels demonstrate superior effectiveness compared to CS-C hydrogels alone in reconstructing hyaline cartilage. Lin et al. [108] prepared a composite hydrogel scaffold using COL, carboxymethyl CH, and Arg-Gly-Asp peptide as raw materials, which performed well in cell adhesion and biocompatibility experiments, and could effectively improve the adhesion of BMSCs on the scaffold, and showed excellent cartilage regeneration.

Polyethylene glycol (PEG) hydrogels are highly attractive scaffold materials for tissue engineering because of their high-water absorption, non-toxicity, and efficient nutrient transport. To improve the interaction between cells and PEG hydrogels, Yang et al. [109] designed cysteine-arginine-glycine-aspartic acid (CRGD), a cell adhesion peptide covalently crosslinked to PEG hydrogels via Michael addition reaction. Their results showed that CRGD improved the interaction between PBMSCs and PEG hydrogels. PEG hydrogels modified with 1 mm CRGD optimally promoted chondrogenic differentiation, induced macrophage polarization toward the M2 type, and facilitated tissue regeneration and repair. Another study confirmed that double network gels composed of polyethylene glycol-chitosan-kartogenin effectively promoted chondrogenic differentiation and viability of PBMSCs, ultimately supporting the regeneration of ACdefects [110] (Table 2).

**Table 2:** Summary and properties of polymers for cartilage tissue engineering.

Polymers		Source or monomer	Advantages	Disadvantages	Ref.
**Natural matrix materials**	Hyaluronic acid (HA)	Animal tissues, bacteria	1. Intimacy with the cell surface 2. Higher stiffness but lower shear than natural nucleus pulposus 3. Preparation of hydrogels by addition, polycondensation, or photo cross-linking	1. Biodegradation in the body by hyaluronidase 2. Poor biomechanical strength 3. Low biodegradability in the crystalline phase	[79,[102], [103], [104]]
	Fibrin	Fibrinogen	Excellent mechanical resistance, elasticity, and web properties	1. Quick rate of degradation 2. Poor biomechanical strength	[111,112]
	Gelatin and Collagen	Animal tissues	1.Superior biocompatibility 2. Favor cell adhesion, proliferation, and extracellular matrix secretion 3. Used as an adjunct to improve the biomechanical and metabolic characteristics of the primary material	1.Low stability under physiological conditions 2. variability degradation	[70,76,[113], [114], [115], [116]]
	Chondroitin sulfate (CS)	Sulfated glycosaminoglycans	1. Safety and excellent biocompatibility 2. Retaining water gives cartilage a high compressive strength and helps lubricate the joints	Pure chondroitin sulfate scaffolds are susceptible to degradation	[74,106]
	Chitosan (CH)	Exoskeletons of crustaceans and insects	1. Chitosan and hydrogels based on chitosan are thermally responsive 2. Has the ability to help hyaline cartilage resist shear and compression forces	1. Poor mechanical strength and stability 2. Not very good cell adhesion 3. Low solubility 4. Quick rate degradation in vivo	[68,107]
**Synthetic materials**	Polyethylene glycol (PEG)	Ethylene glycolEthylene oxide	1. Copolymers or composites to be used for the improvement of their mechanical characteristics 2. Great elastic and bioadhesive	1. Low cellular biorecognition capacity 2. Creates insoluble networks	[109,110]
	Poly(ε-caprolactone) (PCL)	ε-caprolactone	1.High biocompatibility and low immunogenicity 2.Excellent mechanical properties 3. Controls cell growth and vascularization 4. Long and controlled degradation time 5. Available for human body	Low bioactivity	[116]
	Polylactic acid (PLA) and polyglycolic acid (PGA)	Lactide/Glycolide	1.High stress resistance ，biocompatibility and low immunogenicity 2.Excellent mechanical properties 3.Possibility of synthesis in various forms 4. Available for human body	1. Overheating required for depolymerization 2. Localized acidosis due to biodegradation products	[[117], [118], [119]]

In rabbit models of AC defects in the knee joint, Yang et al. used a treatment regimen in which human umbilical cord wharton's jelly co-cultured with chondrocytes, and after 6 months, human umbilical cord wharton's jelly showed faster and better cartilage repair compared with hydrogel. This result provides a new candidate material for regenerative medicine to repair AC defects.

## 6 Cytokines and growth factors in cartilage regeneration

### 6.1 Role of cytokines and growth factors in cartilage development and repair

Cytokines and growth factors are bioactive peptides that play crucial roles in promoting cell growth, proliferation, and chondrogenesis, thus enhancing cartilage repair [120]. These endogenous molecules act through autocrine and paracrine mechanisms in coordination with chondrocytes [121]. Notable cytokines involved in cartilage regeneration include the transforming growth factor (TGF) superfamily, fibroblast growth factor (FGF), insulin-like growth factor 1 (IGF-1), and platelet-derived growth factor (PDGF).

#### 6.1.1 Transforming growth factor superfamily

The TGF-β superfamily, comprising members such as TGF-β1, 2, and 3 as well as bone morphogenetic protein (BMP)-2, 7, and 14, is widely used to induce chondrogenic differentiation and stimulate cartilage ECM production [122]. TGF-β1 is a multifunctional chondroblast growth factor that promotes the synthesis and secretion of proteoglycan and COL2 by chondrocytes [123]. Additionally, TGF-β1 regulates various biological processes, such as cell proliferation, survival, differentiation, migration, and ECM production [124,125]. It also plays a crucial role in maintaining homeostasis between subchondral bone and AC [123]. Studies have shown that active TGF-β1 released by osteoclasts during bone resorption can induce BMSCs to migrate to the site of bone resorption, increase the expression of Sox-9, and enhance ECM production to form new cartilage [126,127].

TGF-β2 shares a biological function similar to TGF-β1 and can regulate chondrogenic and osteogenic differentiation [128,129]. Treatment of dedifferentiated chondrocytes with TGF-β2 led to the re-expression of glucosamine and COL2, restoring the chondrogenic phenotype [128]. Additionally, precursor cells from the perichondrium exhibited chondrogenic differentiation potential when cultured with TGF-β2 and IGF-1, followed by cartilage matrix production [128,130].

Another critical member of the TGF family, TGF-β3, plays a crucial role in cartilage growth and reconstruction by facilitating chondrocyte proliferation, differentiation, and ECM formation [131,132]. It also inhibits various inflammatory mediators, including IL-1, MMPs, and TNF-α, reducing the immune response [131,132]. In vitro studies using a TGF-β3 cross-linked scaffold promoted MSC proliferation and substantial ECM production, while animal models demonstrated MSC chondrogenic differentiation [133].

BMP-2 is an important growth factor for osteochondral tissue regeneration and is capable of inducing cartilage and bone formation [134]. MSCs wrapped with gellan hydrogels and BMP-2 increased ECM production and Sox-9 expression compared to MSCs alone in vitro [135]. Moreover, BMP-2 can reverse chondrocyte dedifferentiation, as evidenced by increased synthesis of cartilage-specific COL2 in dedifferentiated OA chondrocytes [136].

BMP-7, also termed osteogenic protein-1, is a growth factor found in normal AC that stimulates chondrocytes proliferation and differentiation and enhances matrix assembly [137]. Several in vitro studies have shown that BMP-7 stimulates chondrogenic differentiation while promoting ECM synthesis and retention within AC [138,139]. Jelic et al. [140] delivered BMP-7 via a micro-osmotic pump in a sheep model and after 6 months the BMP-7 group showed good filling of defects and regenerated cartilage showed high levels of proteoglycans and COL2.In addition, BMP-7 helps to stimulate cartilage matrix synthesis and works synergistically with other anabolic growth factors, as well as suppressing catabolic factors such as MMP-1, MMP-13, IL-1, IL-6, and IL-8 [120].

BMP-14, also known as growth differentiation factor-5, is a potent chondro-inductive cytokine that promotes endochondral bone growth and chondrogenesis [141]. Transfection of ADSCs with BMP-14 gene resulted in sustained expression of BMP-14, and application of HA hydrogel coated BMP-14-ADSCs to cartilage defect facilitated chondrogenic differentiation of ADSCs and cartilage defect repair [80].

#### 6.1.2 Fibroblast growth factor

The FGF family of growth factors is regulated by perlecan, a heparan sulfate proteoglycan in the ECM, which acts through FGFR-1 and FGFR-3 receptors in chondrocytes [142,143], and functions as a mechanosensor in AC [144].

Two specific members of the FGF family, basic FGF (bFGF, also known as FGF-2) and FGF-18, play prominent regulatory roles in cartilage matrix homeostasis [145]. FGF-2 is known for maintaining the self-renewal and undifferentiated state of human pluripotent stem cells [146], and it has a potent anabolic effect on cartilage homeostasis, making it promising for cartilage regeneration and repair [145]. Hiraide et al. [147] demonstrated cartilage repair in an in vivo model of knee joint degeneration in rabbits by delivering the FGF-2 gene into synovial tissue using an adeno-associated virus. Deng et al. [113] used FGF-2-loaded gelatin microspheres for controlled and sustained release, stimulating the repair of knee cartilage defects in rabbits. The study showed that the areas with previous cartilaginous defects were filled with histologically hyaline-like cartilage after 24 weeks, indicating the potential of FGF-2 scaffolds to promote chondrogenesis.

FGF-18, which is highly expressed in the superficial zone of AC, stimulates the expression and accumulation of COL2 and proteoglycan in articular chondrocytes [148,149], preserving cartilage phenotype and thickness to protect AC against degeneration [112]. DePhillipo et al. [150] confirmed significant improvement in cartilage healing at 6 months after microfracture or osteochondral defect repair in preclinical randomized controlled trials with the use of FGF-18.

#### 6.1.3 Insulin-like growth factor 1

IGF-1 is an essential anabolic factor in AC regeneration [151]. It plays a crucial role in increasing proteoglycan and COL2 synthesis in vitro [151,152] while also regulating the transcription of matrix metalloproteinases to suppress matrix degradation [153]. Moreover, IGF-1 has shown effectiveness in combination with other growth factors and cytokines [154]. When combined with TGF-β, IGF-I enhances the production of cartilage matrix components, including proteoglycan, and upregulates the expression of COL2 and aggrecan genes in chondrocytes [155], thereby promoting the chondrogenic differentiation of MSCs [156]. In vitro research, An et al. [157] demonstrated that IGF-1 in combination with BMP-2 could direct ADSCs toward chondrogenic differentiation.

#### 6.1.4 Platelet-derived growth factor

PDGF is a chemotactic factor and stimulator for chondrocytes and MSCs, facilitating cartilage formation by increasing proteoglycan production and chondrocytes proliferation [158]. It also promotes chondrogenic differentiation of BMSCs and enhances chondrocytes metabolism [159]. Additionally, PDGF has been proven to inhibit IL-1β induced cartilage degradation by suppressing NF-κB signaling [120].

### 6.2 Signaling pathways involved in cartilage regeneration

**Fig. 2:**
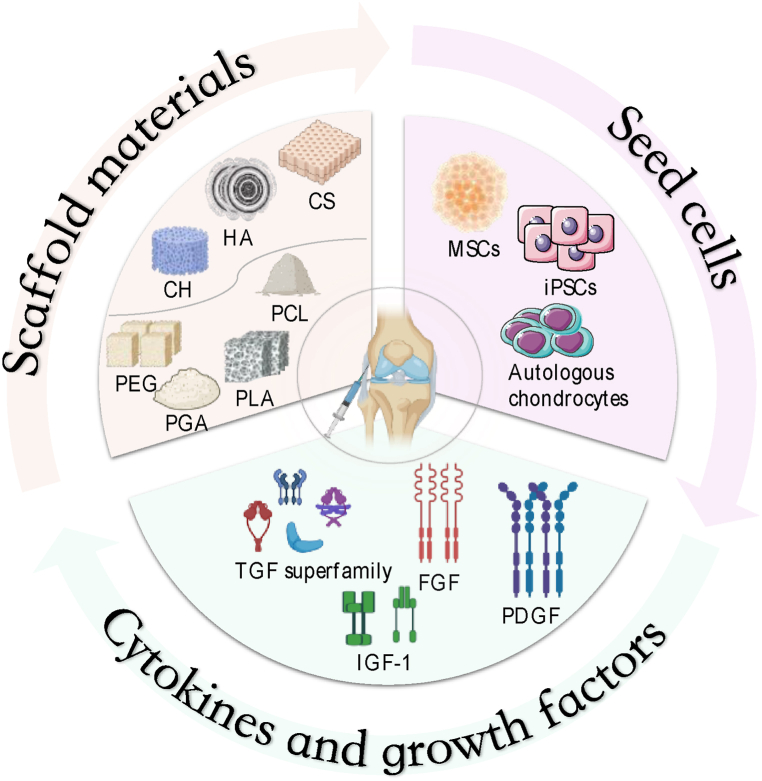
Overview of the three core elements of tissue engineering for cartilage repair. Currently, seed cells can be categorized into autologous chondrocytes, mesenchymal stem cells (MSCs), and induced pluripotent stem cells(iPSCs). Scaffold materials can be broadly categorized into natural matrix materials and synthetic materials. Commonly used natural matrix materials include hyaluronic acid (HA), chondroitin sulfate (CS), and chitosan (CH). Commonly used synthetic materials include polyethylene glycol (PEG), poly(ε-caprolactone) (PCL), polylactic acid (PLA), and polyglycolic acid (PGA). Cytokines and growth factors include the transforming growth factor (TGF) superfamily, fibroblast growth factor (FGF), insulin-like growth factor 1(IGF-1), and platelet-derived growth factor (PDGF).

Many members of the TGF-β superfamily are potent inducers of chondrogenesis. TGF-β1 and TGF-β3 exhibit higher potency in mediating this process than TGF-β2 [160]. BMP signaling is essential for chondrogenic differentiation, as Sox protein expression relies on BMP signaling through the serine/threonine kinase receptors ALK3 and ALK6, mediated by the classical Smad pathway [161,162]. Among BMPS, BMP-2 has been shown to have a particularly strong stimulating effect on cartilage formation [160], while BMP-7 promotes ECM synthesis and inhibits catabolic factors [120]. BMPs can also activate mitogen-activated protein kinase (MAPK) signaling through non-Smad-dependent pathways, including the p38 and ERK1/2 cascades, as well as the Ras/ERK1/2 or RhoA/ROCK axis [161,163,164]. These pathways play critical roles in chondrogenic differentiation by activating target transcription factors such as AP-1, ETS, Runx2, HIF-2α, and C/EBPβ [165]. Additionally, several other serine/threonine protein kinases (e.g., PKA [166], PKC [167], Rho kinase [160]) and phosphoprotein phosphatases (e.g., PP1, PP2A, calcineurin [168]) have been identified as key regulators of chondrogenesis, exerting either stimulatory or inhibitory effects. The Notch pathway is also active during the early stages of chondrogenesis, where transient signaling is necessary for initiating MSCs differentiation but must be switched off in subsequent stages [169](Fig. 2).

### 6.3 Delivery systems for cytokines and growth factors

Biopolymer injectable hydrogels serve as crucial carriers and scaffold materials for tissue engineering, enabling sustained release of specific cytokines. Zheng et al. [170] developed a polymeric silk-polylysine-modified chitosan polymer derived from thermosensitive glycerol phosphate, which continuously releases TGF-β1 and effectively regulates the expression of cartilage-specific and inflammation-related genes. Mao et al. [171] designed a cartilage-biomimetic silk fibroin-based scaffold with specific affinity peptide and controlled sequential release of TGF-β1, creating an ideal 3D microenvironment for cartilage reconstruction. Yang et al. [75] loaded BMSCs onto gelatin methacrylate-based hydrogel microspheres with superwettability and continuous release of TGF-β, effectively promoting cartilage regeneration.

### 6.4 Strategies to enhance bioactivity and release kinetics

Enhancing biological activity and optimizing the kinetics of therapeutic agent release are essential strategies to improve the effectiveness of bioactive molecules and growth factors, leading to successful cartilage repair. In this section, we will discuss several explored approaches for achieving these goals.

#### 6.4.1 Modification of biological materials

In cartilage tissue engineering, Biomaterials can be modified to enhance biological activity and control the release of therapeutic agents. Surface modifications, such as incorporating specific peptides or chemical groups, promote cell adhesion, proliferation, and differentiation [117]. For example，Mao et al. [171] developed a cartilage-biomimetic silk fibroin-based scaffold with excellent structural stability and cartilage-like mechanical properties, providing an ideal microenvironment for cartilage reconstruction. Optimizing the interaction between cells and scaffolds can be achieved through surface modifications of biomaterials, enhancing biological activity and tissue integration. Furthermore, incorporating bioactive molecules into the biomaterial matrix enables sustained release, prolonging their availability and therapeutic effects [106]. This strategy enhances the performance of biomaterials in cartilage tissue engineering and improves the potential for successful cartilage regeneration.

#### 6.4.2 Controlled release system

Controlled-release systems have revolutionized therapeutic agent delivery, allowing precise temporal and spatial control. These systems involve encapsulating biologically active molecules within carriers such as hydrogels or microspheres, ensuring sustained and controlled release over time [75,172]. For example, Zheng et al. [170] utilized a silk fibroin poly(l-lysine)-modified chitosan polymer (SF/PCS) from thermosensitive glycerol phosphate. The SF/PCS hydrogel group continuously released TGF-β1, effectively regulating cartilage-specific and inflammation-related genes and promoting tissue regeneration in vitro and in vivo. Another study by Yang et al. [75] loaded BMSCs onto gelatin methacryloyl hydrogel microspheres with superwetting properties, enabling sustained release of TGF-β and promoting cartilage regeneration at bone defect sites. By manipulating factors such as carrier material properties, degradation rates, and cross-linking mechanisms, desired release kinetics can be achieved [94,173]. This approach ensures sustained and localized delivery of therapeutic agents, enhancing their biological activity and facilitating targeted cartilage regeneration. For example, Jelic et al. [140] delivered BMP-7 to the cartilage defect area of a sheep model using micro-osmotic pumps, resulting in high levels of proteoglycan and COL2 in the regenerated cartilage area after 6 months.

#### 6.4.3 Combination therapy

Combining various bioactive molecules or growth factors has shown promising effects in promoting cartilage regeneration. For instance, in a rabbit cartilage defect model, the polylactide-*co*-glycolide/fibrin gel scaffold containing MSCs plus TGF-β1 exhibited higher gene expression profiles of COL2, aggrecan, and Sox9 compared with the scaffold containing MSCs alone [111]. Synergistic effects can be achieved by using a combination of growth factors with complementary functions. Simultaneous delivery of TGF-β and BMP, for example, can promote chondrogenesis and ECM synthesis, resulting in improved cartilage repair [174]. The combined application of IGF-1 and BMP-2 allows ADSCs to differentiate into chondrocyte-like cells [155]. Therefore, the combined treatment approach can improve the biological activity and is expected to offer a more comprehensive and effective strategy for cartilage regeneration.

#### 6.4.4 Biophysical stimulation

Biophysical stimulation techniques, including mechanical force, electrical stimulation, and ultrasound, have been studied to enhance biological activity and optimize the release kinetics of therapeutic agents [175,176]. These techniques promote cellular responses such as cell proliferation, differentiation, and ECM synthesis, studied to enhance biological activity and optimize the release kinetics of therapeutic agents [177].

## 7 Tissue engineering approaches for articular cartilage regeneration

### 7.1 Cell-seeded scaffolds for cartilage tissue engineering

#### 7.1.1 Scaffold seeding methods

The structure and microstructure of scaffolds significantly influence their function in vitro and in vivo, as well as their potential clinical applications in tissue engineering research. A well-connected pore structure promotes uniform cell distribution, adhesion, and efficient diffusion of nutrients and waste products within the scaffold [178]. Polylactic acid-glycolic acid copolymer (PLGA) is a commonly used scaffold composed of polyglycolic acid (PGA) and polylactic acid (PLA). PLGA offers controllable biodegradability, low immunogenicity, and the ability to deliver drug carriers to target tissues [117]. Tang et al. [118] utilized 3D printing technology to combine a rigid polylactic acid-hydroxyacetic acid scaffold with platelet-rich plasma (PRP) hydrogel, achieving efficient MSCs delivery and growth factors release for tissue healing regulation [119]. PLGA combined with cytokine implantation, such as TGF-β3 or SDF-1α, has shown enhanced MSC residency and differentiation into AC. However, the clinical application of PLGA for AC repair remains experimental [117].

#### 7.1.2 Strategies to promote cell attachment, proliferation, and differentiation

Genetic engineering techniques can be employed to enhance the delivery of biological agents within the joint [179]. Zhang et al. [180] developed a methacrylated gelatin-chitosan composite hydrogel loaded with kartogenin, which promoted in situ cartilage differentiation, gene expression, and glycosaminoglycan deposition, facilitating cartilage tissue regeneration. Furthermore, the results showed that a gelatin methacrylate/affinity peptide sequence PFSSTKT-modified ECM hydrogel scaffold could rapidly recruit endogenous stem cells and induce BMSC chondrogenic differentiation [114]. Overexpression of platelet-derived growth factor in MSCs, achieved through genetic modification, can recruit endogenous stem/progenitor cells to injured tissues, stimulating MSCs chondrogenic potential and anti-inflammatory properties [181]. MIR410 has been shown to regulate chondrogenic differentiation and subchondral bone remodeling through the Wnt signaling pathway [182]. Pei et al. demonstrated that a gelatin methacryloyl-BMSC scaffold, combined with upregulated MIR410, promoted migration, proliferation, and chondrogenic differentiation of MSCs for AC regeneration [70,76]. Other studies have explored candidate genes like SCRG1, which inhibits Wnt/β-catenin signaling and promotes chondrogenic differentiation in UCMSCs [183]. Additionally, TGF-β1/RADA-16 hydrogel accelerated chondrogenic differentiation and glycosaminoglycan production in BMSCs [184], while integrin α10-MSCs differentiated into chondrocytes and produced cartilage matrix in vivo [77].

Nanomaterials have been utilized to enhance scaffold performance through their nanobiological effects and responsiveness to external stimuli. These strategies have demonstrated superior outcomes in chondrogenic gene expression, matrix deposition, and cartilage repair. For instance, tetrahedral framework nucleic acids exhibit promise in enhancing cell proliferation, migration, and cartilage regeneration [47]. Graphene oxide, a derivative of graphene with oxygen-containing functional groups, can be bound to hydrogels and has been shown by Liu et al. [185] to promote chondrocytes secretion, reduce joint inflammation, and facilitate AC repair. Another effective approach involves the delivery of kartogenin using multifunctional Prussian blue nanocomposites developed by Liu et al. [173]. This method promotes chondrogenic differentiation while inhibiting excessive MSC differentiation. In a rat cartilage defect model, precise and controlled release of this nanomaterial through intra-articular injection, triggered by near-infrared, achieves excellent cartilage repair [173]. Additionally, Cai et al. [186] prepared bionic copper sulfide@phosphatidylcholine nanoparticles loaded with TGF-β1 plasmid DNA. These nanoparticles exhibited superiority over pure MSCs in terms of chondrogenic gene expression, glycosaminoglycan deposition, and COL2 formation. Intra-articular administration of these nanoparticles significantly enhanced the repair of damaged cartilage [186].

### 7.2 Scaffold-free approaches

#### 7.2.1 Self-assembled constructs

Other naturally sourced hydrogel materials, such as decellularized extracellular matrix (dcECM) and self-assembling peptides, have shown potential for cartilage repair and regeneration. DcECM is a biomaterial that requires decellularization while preserving the essential characteristics of the extracellular matrix, resembling natural tissues and providing a suitable environment for cellular recognition. Studies have demonstrated that dcECM-derived hydrogels exhibit good biocompatibility, appropriate physicochemical and mechanical properties, and the ability to induce cartilage formation in MSCs and promote hyaline cartilage-like tissue formation after implantation [187]. For example, graphene oxide-modified 3D dcECM scaffolds prepared by Gong et al. [78] showed enhanced cell adhesion, proliferation, and differentiation in vitro. When loaded with BMSCs, these composite scaffolds successfully bridged rabbit knee cartilage defects with hyaline cartilage after 12 weeks of implantation. Yuan et al. [188] prepared bioactive magnesium-containing glass nanospheres (Mg-BGNs) composite scaffolds by doping bioactive magnesium-containing glass nanospheres into dcECM, which stimulated both AC and subchondral bone regeneration, providing a potential option for cartilage/bone regeneration. UCMSCs loaded with a graphene oxide particle lubricant reduced inflammation levels and improved the biochemical environment in the joint cavity, thereby promoting AC repair [189]. Another approach utilized a self-assembled peptide, Ac-(RADA)_4_-CONH_2_, loaded with LIANAK to create a TGF-β1-mimicking hydrogel peptide. The LIANAK-functionalized hydrogel peptide showed good bioactivity, structural stability, contributed to gene expression and ECM deposition for cartilage formation [172]. Qu et al. [190] reported an injectable, biodegradable photo-crosslinked acacia bean gum hydrogel based on a natural polysaccharide polymer-methacrylate hydrogel. This innovative treatment accelerated the healing of damaged cartilage in vivo for minimally invasive cartilage repair procedures [190].

#### 7.2.2 Bioprinting techniques

3D printing technology, also known as additive manufacturing, enables precise control over the composition and spatial distribution of cells and biomaterials to create scaffolds for tissue repair and regeneration. It involves stacking biologically active materials filled with cells and growth factors layers to create highly biomimetic tissue microenvironments, structures, blood vessels, and functional artificial organs. Yan et al. [191] demonstrated that Exos cultured in a 3D environment have a stronger regulatory ability in the articular cavity microenvironment compare to those derived from MSCs cultured in a 2D environment. In a rat knee AC defect model, the use of 3D-exos combined with subsequent articular cavity injections showed superior cartilage repair, reduced inflammation, and promoted macrophage polarization toward the M2 phenotype.

Gelatin, derived from natural collagen, closely mimics the ECM of AC, providing a favorable microenvironment for implanted cells in 3D-printed scaffolds. Huang et al. [115] utilized a mixture of gelatin and hydroxyapatite as a scaffold for human umbilical cord blood MSCs (hUCB-MSCs) by microextrusion 3D bioprinting and enzymatic cross-linking methods. This approach supported the hUCB-MSCs adhesion, growth, proliferation, and induced their chondrogenic differentiation in vitro. Yang et al. [116] developed printing inks containing ECM, methacrylate gelatin, and TGF-β3 encapsulated polylactic-coglycolic acid microspheres, which were coprinted with poly(ε-caprolactone). The scaffold achieved continuous release of TGF-β3, directing endogenous stem/progenitor cell migration and differentiation. In a sheep model, this approach demonstrated excellent tissue repair and recapitulated the anisotropic structure of natural AC. Guan et al. [192] prepared a novel photo-crosslinkable printable consisting of polyethylene glycol diacrylate, methacryloyl gelatin, and chondroitin methacrylate sulfate for printing 3D scaffolds for cartilage tissue regeneration. The 3D-printed scaffold exhibited sufficient mechanical strength, compressive elastic modulus, degradation rate and maintained the 3D microenvironment necessary for BMSC differentiation, proliferation, and ECM generation. These findings suggest that this customizable 3D-printed scaffold holds great potential for in vivo cartilage repair and regeneration. Olmos-Juste et al. [193] developed functional alginate and waterborne polyurethane scaffolds using 3D technology. After 28 days of in vitro cartilage formation experiments, the scaffolds with alginate contents of 3.2 % and 6.4 %, respectively, were able to synthesize up to 6 μg of glycosaminoglycan specialized ECM, which showed excellent performance in regenerating AC tissue in vitro.

### 7.3 Bioreactor systems for cartilage tissue engineering

#### 7.3.1 Importance of mechanical stimulation

Mechanical stimulation plays a crucial role in AC generation and maintenance. Bioreactor systems provide specific mechanical stimuli that can enhance chondrogenic properties in AC tissue engineering using human cells [176]. Semitela et al. [194] demonstrated that mechanical stimulation significantly improves cell metabolic activity and ECM deposition. In vivo, mechanical loading is a vital factor for successful cartilage regeneration. Researchers have developed mechanobiological devices that provide mechanical stimulation to mimic this loading. Li et al. [195] developed a knee joint loading device that promoted the migration and chondrogenic differentiation of MSCs, leading to cartilage defect repair. Ouyang et al. [196] demonstrated that appropriate cyclic sinusoidal dynamic stretching improved the proliferative capacity and cartilage phenotype of chondrocytes through coculturing with MSCs.

#### 7.3.2 Perfusion and compression bioreactors

Perfusion bioreactors utilize perfusion media to enhance cell seeding and promote glycosaminoglycan synthesis and deposition in chondrocytes and MSCs, surpassing static culture [197]. Culturing MSCs in perfused conditions within biomaterial scaffolds improves cell survival, enhances ECM deposition, and facilitates osteoblast differentiation [198]. Mitra et al. [199] reported that constructs maintained in perfusion conditions for at least 14 days exhibited improved bone formation in vivo [197]. Moreover, perfusion bioreactors have proven effective in generating vascularized bone tissue grafts, which are critical for graft survival post-implantation. Perfusion bioreactors provide dynamic microfluidic environments that positively influence cellular responses. Fluid shear stress, an important part of the body microenvironment, plays a crucial role in the development of bone-like tissues in a 3D cellular network. Increased fluid shear stress increases mineral deposition, promotes osteoblast phenotypes, and improves the spatial distribution of the ECM within the porous 3D scaffolds [200].

Compression bioreactors, designed to simulate natural physiological loading, are increasingly employed in bone and cartilage tissue engineering [115]. Dynamic compression has been shown to enhance the functional properties of chondrocytes constructs in cartilage tissue engineering. Hoenig et al. [201] subjected MSCs constructs to dynamic compressive loading for 6 weeks and observed improved mechanical properties of the cartilage structure. Mauck et al. [202] applied dynamic compression to load cell agarose discs at physiological strain levels for 4 weeks, resulting in increased chondrocytes matrix production and the formation of functional cartilage tissue structures. Compressive bioreactors also improve glycosaminoglycan formation, hydroxyproline content, and elastic modulus, closely resembling natural cartilage [203,204]. Sittichockechaiwut et al. [205] demonstrated the high sensitivity of osteoblasts to mechanical loading, as even short periods of compressive loading had a strong effect on the mineralized matrix generation and osteogenesis-related genes expression. Quantitative mechanical stimulation increases the gene expression of chondrogenic markers, such as Sox-9, COL2 and aggrecan, in cultured MSCs [177].

#### 7.3.3 Bioreactor-mediated cell culture strategies

Perfusion-based cell culture bioreactors have shown superior cavity surface coverage compared to static injection, thus enhancing the quality of engineered tissues by enabling reproducible and controllable changes in specific environmental factors. By automating and standardizing tissue fabrication in a controlled closed system, bioreactors reduce production costs, facilitating broader applications of engineered tissues [206]. Perfusion-based cell seeding methods improve the uniformity of the initial cell distribution on porous scaffolds for various tissue engineering applications [207].

**Fig. 3:**
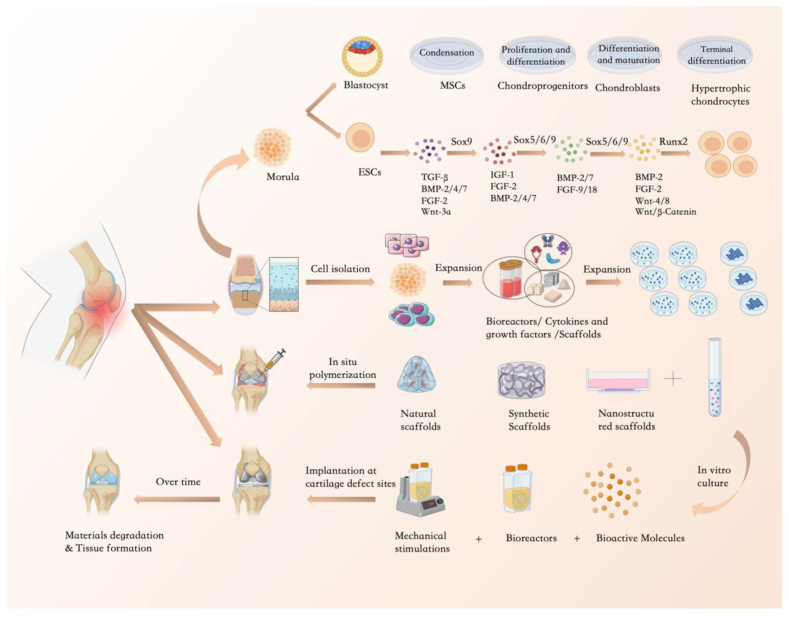
Overview of a schematic diagram of tissue engineering for repair of cartilage defects and a sequence of events for inducing chondrogenic differentiation of mesenchymal stem cells. Tissue engineering methods include cell culture, scaffold implantation, different biomaterials and in vitro culture. The different stages and treatments of cartilage formation as well as the cell culture process are represented schematically and the major growth factors involved in each step are listed in the figure.Term: MSCs:mesenchymal stem cells; ESCs: embryonic stem cells; TGF:transforming growth factor; FGF: fibroblast growth factor; IGF: insulin-like growth factor; BMP: bone morphogenetic protein.

Currently, bioreactors for cell-loaded cultures incorporate fluid shear, liquid pressure, and cell-direct solid pressure loading devices [208]. Onal et al. [209] reviewed the progress in mechanical compression of living cells using microdevices. Compressive stress plays a vital role in cell growth, differentiation, migration and invasion [210]. Solid stress allows the study of changes in cell morphology and behavior induced by external static or dynamic compression [211]. Microfluidics provides a useful tool for simulating the in vivo microenvironment in a microarray culture system and controlling the application of compressive forces to individual cells in both 2D and 3D culture models [209,212](Fig. 3).

Summary of regenerative strategies regarding cartilage repair, including scaffold seeding, cytokines and growth factors, self-assembled constructs, bioprinting techniques, genetic engineering, bioreactor system, bioactivity and release kinetics, and cell delivery strategies. Among them, bioactivity and release kinetics include modifying biological materials, employing controlled release system, and utilizing combination therapy. The bioreactor system includes mechanical stimulation, perfusion and compression. Scaffold materials include hyaluronic acid (HA), chondroitin sulfate (CS), chitosan (CH), and polyethylene glycol (PEG). Seed cells for articular cartilage regeneration include autologous chondrocyte, mesenchymal stem cells (MSCs), and induced pluripotent stem cells (iPSCs). Crucial cytokines and growth factors involved in cartilage regeneration include members of the transforming growth factor (TGF) superfamily, fibroblast growth factor (FGF), insulin-like growth factor 1 (IGF-1), and platelet-derived growth factor (PDGF).

## 8 Challenges and future perspectives

Tissue engineering aims to create tissues that mimic normal AC in biomechanics, biochemistry, and histocytology by integrating seed cells, scaffolds, and cellular factors [15]. Gene therapy has shown promise in introducing growth factors to target cells and facilitating stable and continuous repair in injured cartilage. Although animal experiments have yielded positive results, further investigation is required to assess their clinical safety, reliability, and efficacy [213]. However, challenges persist in the repair process. For instance, the repaired tissue often fails to fuse properly with adjacent normal cartilage, leading to high shear stress, degeneration, and even detachment, ultimately resulting in repair failure [2]. Natural polymer hydrogels have attracted significant attention from material scientists due to their advantages, including high biocompatibility, degradability, and low inflammatory response. These hydrogels show promise in facilitating the development of seed cells, making them more suitable for cartilage therapy and offering a potential therapeutic platform for cartilage regeneration. Nevertheless, challenges such as poor mechanical properties, inadequate cell adhesion, and limited long-term stability hinder their clinical translation [214].

Despite some successes in stem cell-based cartilage tissue engineering, their clinical translation for cartilage repair and regeneration remains severely limited. Ethical and regulatory concerns aside, there are various barriers to the translation of stem cell products, such as the availability of preclinical data, manufacturing and facility costs, and government regulations [215]. Ethical considerations also play a role in tissue engineering research, encompassing animal protection, human embryo sourcing, and histocompatibility of scaffold materials. In clinical applications, issues related to safety, effectiveness, and patients' right to information need to be addressed. Therefore, tissue engineering regenerative medicine should be employed in clinical practice with strict adherence to scientific research design, careful selection of seed cells and scaffold materials, and a thorough understanding of safety and effectiveness, all while respecting patients' rights.

## 9 Conclusion

Tissue engineering is an interdisciplinary field that combines biology, engineering, materials science, and surgery to address the challenges of tissue regeneration. It comprises three essential components: seed cells, scaffold materials, and cytokines. The use of biological scaffolds in cartilage tissue engineering aims to create a suitable microenvironment for cells-mediated synthesis of cartilage matrix and temporary restoration of cartilage function until new tissue formation occurs. However, cartilage tissue has limited regenerate capacity due to factors such as the absence of blood and nerve supply and cellular homogeneity. The advent of tissue engineering has opened up possibilities for cartilage regeneration, with scaffold materials playing a crucial role.

Natural polymer materials derived from polysaccharides, proteins, and other sources. have shown excellent biocompatibility, degradability, and unique self-healing properties, offering new directions for cartilage defect repair. Nevertheless, challenges remain in translating tissue engineering into clinical practice, including issues such as inadequate mechanical properties, limited cell adhesion, insufficient long-term stability, and the presence of pathological microenvironment in degenerative cartilage injury. Overcoming these obstacles and continually improving and innovating biomaterials and therapeutic approaches in tissue engineering hold great promise for advancing the clinical treatment of cartilage defects.

## Author contribution statement

Maohua Chen: Writing – original draft, Conceptualization. Zhiyuan Jiang: Supervision, Conceptualization. Zhen Cai: Supervision. Jinming Huang: Writing – review & editing, Visualization. Xiuyuan Zou: Writing – original draft. Xiaobo You: Conceptualization

## Funding statement

This work was financially supported by the China postdoctoral science foundation (2023M732442 to Jinming Huang), the Key R&D Projects of Sichuan Science and Technology Department (2022YFS0155 to Xiaobo You), and the Youth Talent Fund of Sichuan Provincial People's People's Hospital (2022QN58 to Zhiyuan Jiang).

## Additional information

No additional information is available for this paper.

## Declaration of competing interest

The authors declare that they have no known competing financial interests or personal relationships that could have appeared to influence the work reported in this paper.
